# Dispersion Interactions in Exciton-Localized States. Theory and Applications to π–π* and n−π* Excited States

**DOI:** 10.1021/acs.jctc.2c00221

**Published:** 2022-05-19

**Authors:** Mohammad Reza Jangrouei, Agnieszka Krzemińska, Michał Hapka, Ewa Pastorczak, Katarzyna Pernal

**Affiliations:** † Institute of Physics, Lodz University of Technology, ul. Wolczanska 217/221, 93-005, Lodz, Poland; ‡ Faculty of Chemistry, University of Warsaw, ul. L. Pasteura 1, 02-093, Warsaw, Poland

## Abstract

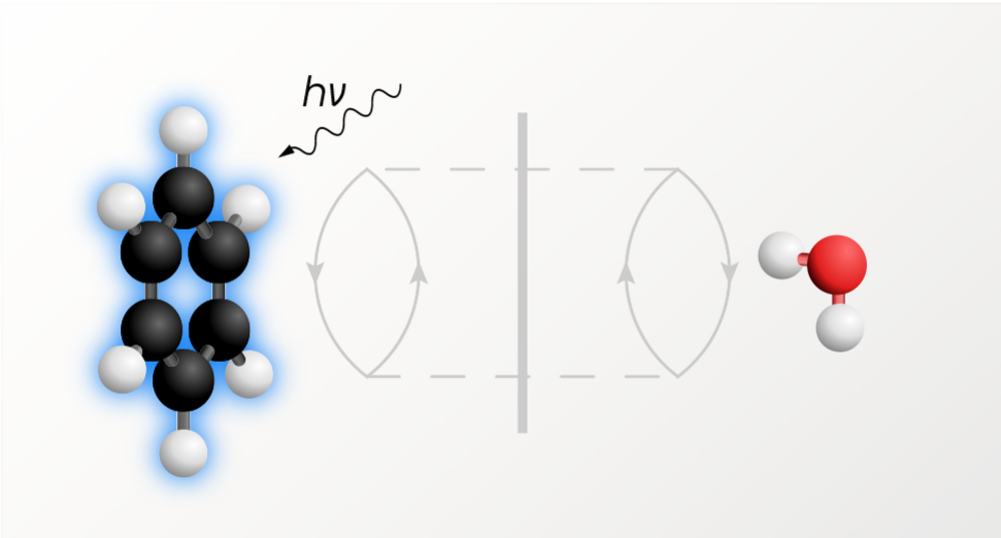

We address the problem of intermolecular interaction energy calculations in molecular complexes with localized excitons. Our focus is on the correct representation of the dispersion energy. We derive an extended Casimir-Polder formula for direct computation of this contribution through second order in the intermolecular interaction operator *V̂*. An alternative formula, accurate to infinite order in *V̂*, is derived within the framework of the adiabatic connection (AC) theory. We also propose a new parametrization of the VV10 nonlocal correlation density functional, so that it corrects the CASSCF energy for the dispersion contribution and can be applied to excited-state complexes. A numerical investigation is carried out for benzene, pyridine, and peptide complexes with the local exciton corresponding to the lowest π–π* or n– π* states. The extended Casimir-Polder formula is implemented in the framework of multiconfigurational symmetry-adapted perturbation theory, SAPT(MC). A SAPT(MC) analysis shows that the creation of a localized exciton affects mostly the electrostatic component of the interaction energy of investigated complexes. Nevertheless, the changes in Pauli repulsion and dispersion energies cannot be neglected. We verify the performance of several perturbation- and AC-based methods. Best results are obtained with a range-separated variant of an approximate AC approach employing extended random phase approximation and CASSCF wave functions.

## 1 Introduction

The theoretical description of intermolecular forces underlying fundamental physical and chemical phenomena continues to pose a challenge for quantum science. The knowledge of accurate potential energy surfaces gives access to measurable quantities such as rovibrational spectra, phase equilibria, or molecular crystal structures. Although most of the focus so far has been on interactions in ground-state systems, there has been a growing interest in investigating bound molecular systems in excited states.

Interactions involving excited-state molecular species are crucial in fundamental processes of charge^1^ and energy transfer.^2^ Modeling of intermolecular forces is therefore useful in designing nanostructures with high phosphorescence quantum yields^3,4^ or optoelectronic materials.^5,6^ For instance, the efficiency of organic light emitting diodes can be increased by exploiting the spin fission process, in which a highly energetic singlet exciton is converted into two triplet excitons.^7^ Spin fission may be driven by molecular interactions,^8−10^ but the mechanism of this process is not fully understood.^11^ Interactions between an excited molecule and its environment is also an active field of research. There, accurate prediction of the solvent effects on the absorption and emission bands requires going beyond simple electrostatic models.^12,13^

A reliable description of interactions in excited-state complexes is intrinsically more difficult than in the case of ground states. The multireference second-order perturbation approaches, which are the methods of choice for these systems, are plagued with the well-known problems of size-inconsistency and intruder states.^14^ Coupled cluster response theories, such as CC2 and CC3, are a viable alternative,^15,16^ but they are limited in practice to small- and medium-size systems. For larger complexes, time-dependent DFT (TD-DFT) or its semiempirical variants offer the best accuracy-to-cost ratio.^14,17^ Unfortunately, the combination of weak intermolecular forces and excited states is particularly challenging even for modern exchange-correlation functionals. In particular, ground-state semiempirical corrections for the dispersion energy are no longer adequate. They have been parametrized to account for the ground state dispersion energy and may fail miserably if applied to excited states involving redistribution of electron density.^18^ To the best of our knowledge, only the local response dispersion (LRD) model of Nakai and co-workers^19^ has been extended specifically to excited-state systems.^20^ It should be noticed that the LRD approximation is based on assumptions valid for ground states, which may affect the accuracy of the method when applied to excited-state molecules.

In this work, we formulate a framework to describe the London dispersion energy in electronically excited van der Waals complexes. We first generalize the Casimir-Polder formula to excited states. We show that the emergent terms, absent from the ground-state expression, can be positive, which can ultimately lead to repulsive dispersion terms. We then derive an alternative dispersion energy expression by employing the adiabatic correction (AC) theory for multiconfigurational wave functions.^21^ The AC-based formula is correct through infinite order in the interaction operator. A connection between the supermolecular AC energy and the second-order dispersion energy is established in the long-range regime.

The dispersion energy for excited-state complexes is computed using response properties obtained from solutions of extended random phase approximation (ERPA) equations^22^ and wave functions of the complete active space (CAS) type. The damping of the dispersion energy is represented via the exchange-dispersion term calculated in the framework of multiconfigurational symmetry-adapted perturbation theory, SAPT(MC).^23,24^ We assess the performance of several approximate approaches for excited-state interactions based on CAS self-consistent field (CASSCF), which account for the dispersion energy. A special attention is paid to the representation of dispersion forces in the recently developed variants of the AC theory.^21,25,26^ Methods combining wave function and DFT are also investigated, including multiconfigurational range-separated theory^27^ and reparametrization of the nonlocal van der Waals VV10^28^ functional.

Our focus is on noncovalent interactions in complexes of low-lying excited states either of a π–π* or n−π* character. The reason for choosing these systems is 2-fold. First, the π–π* and n−π* excitations are the keystone of organic photochemistry^29^ and of keen interest to computational chemists.^30^ Second, owing to the localization of the exciton, the excimer and resonance phenomena do not overshadow the effect of dispersion.

Several works investigated excited-state complexes of the π–π* and n−π* types. Reimers and Cai^31^ studied changes in the relative stability of hydrogen bonds between heteroaromatic rings and water upon n−π* excitations. The authors observed a weaker binding of the excited-state in linear geometries. In contrast, on-top structures become more stable compared to ground states. Ge and Head-Gordon^32^ performed an energy decomposition analysis (EDA) based on absolutely localized orbitals (ALMOs) for representative pyridine/pyrimidine–water complexes. The decrease in electrostatics following the n−π* transition was identified as the major factor responsible for changes in the binding strength. Similar conclusions were also drawn from density functional tight binding (DFTB) interaction energy decomposition.^33^ Although the rearrangement of the electron density is no doubt the primary effect behind the change from ground- to excited-state interactions, second-order effects in the intermolecular potential cannot be neglected.^34^ At present, neither ALMO- nor DFTB-EDA provides a rigorous account for the second-order dispersion energy. The latter relies on dispersion corrections^35,36^ parametrized for ground-state interactions. The former, ALMO-EDA for excited states, is based on configuration interactions singles (CIS) method, thus missing the bulk of dispersion interactions.^32,37^ An alternative to variational EDAs, which offers a correct description of dispersion interactions, is SAPT.^38^ To complement previous findings, we investigate the character of interactions of n−π* and π–π* states employing the SAPT(MC) variant^24^ of the theory. The method is applied with CASSCF reduced density matrices for the monomers.

The structure of the paper is as follows. In section 2, the second-order expression for the dispersion energy is turned into an extended Casimir-Polder formula valid for complexes with localized excitons, while in section 3, a dispersion energy formula is derived from the adiabatic connection approach. Multiconfigurational methods adequate for computing interaction energies in excited state complexes are presented in section 4. Results for interaction energies in complexes with π–π* and n−π* excitons are presented and discussed in section 5, and the paper is summarized and concluded in section 6.

## 2 Extended Casimir-Polder Formula for Excited States

First we investigate the dispersion energy expression which is purely nonclassical and results from the long-range electron correlation. If a combined system *AB* dissociates into subsystems *A* and *B* in states *I* and *J*, respectively
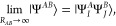
1and the unperturbed state  is not degenerate, then the dispersion energy is well-defined in the Rayleigh–Schrödinger (RS) perturbation theory as a second-order term in the interaction potential reading^39^
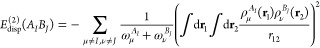
2where a transition density for an *N*_*A*_-electron subsystem *A* corresponding to a *I* → μ state transition, is defined as
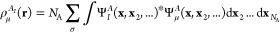
3(analogously for *B*), and **x** = (**r**, σ) combines Cartesian and spin coordinates. It should be mentioned that a formula valid for degenerate-state dimers could be developed by employing a degenerate perturbation theory (see, e.g., refs (40−42) where the authors computed *C*_*n*_ coefficients of homoatomic dimers consisting of a ground-state atom interacting with an excited-state atom).

The dispersion energy formula includes transition energies for monomers between the unperturbed and excited states, that is, for the monomer *A* we have
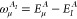
4In general, the formula for the dispersion energy can be divided into four parts
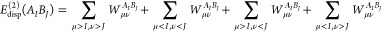
5where
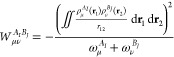
6The first term involves only positively signed up-transition energies for both monomers; that is, 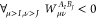
7and it takes a negative sign. The second term in eq 5 does not vanish only if excitons are localized on both monomers, it includes only terms corresponding to negative transitions
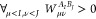
8and is of a positive sign.

What distinguishes the dispersion energy expression pertaining to an excited-state system from that of the ground-state expression (corresponding to *I* = 0 and *J* = 0), is that for the latter all transition energies are positive, while the former includes negative transition energies corresponding to μ < *I*; that is, 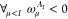
9(analogously for *B*). The presence of negative transition energies modifies the Casimir-Polder formula^43^ for the dispersion energy. To see this, introduce factorization of the denominators involving positive transition energies in eq 2 by employing the integral identity
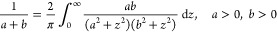
10and express  by means of the density response function of the imaginary frequency, for the noninteracting subsystem *A* in the *I*th state
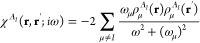
11and that of *B* in the state *J*, , defined analogously. From now on it will be assumed that wave functions are real-valued. Decomposing the response functions of the monomers into positive- and negative-transition-energy components as
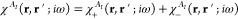
12
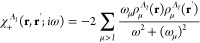
13
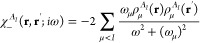
14(analogously for *B*), the dispersion energy can be written as
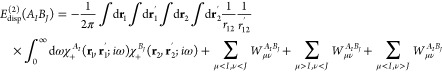
15where the  terms are defined in eq 6. The expression in eq 15 is an extension of the Casimir-Polder formula (also referred to as the Longuet-Higgins formula^44^) for excited states. The ground-state dispersion energy is expressed entirely through χ = χ_+_ response functions. For excited states, also the non-Casimir-Polder terms, , have to be included. They arise due to the presence of negative transitions in the density response function of the unperturbed monomers. While the first term in eq 15 is always negative and attractive,^45,46^ the non-Casimir-Polder terms, eq 6, may take a positive sign. For example, for a system with two localized excitons, one on a subsystem *A*, another on *B*, non-Casimir-Polder terms corresponding to negative transitions on *A* and *B*, μ < *I*, ν < *J* in eq 6, are positive. Thus, this kind of non-Casimir-Polder terms gives rise to repulsion for multiple localized-exciton states.

It is worth noticing that the multipole expansion of non-Casimir-Polder terms is identical as in the case of terms with positive transition—both decay with the sixth power of the inverse of the intermonomer distance *R*_*AB*_. This is in contrast to analogous terms derived from nonrelativistic quantum electrodynamics in the multipolar formalism, fully accounting for the retardation effects.^47^ The non-Casimir-Polder terms obtained in this theory involve contributions associated with the real-photon exchange between molecules, and they fall off only as , i.e., five orders of magnitude more slowly than terms corresponding to positive transitions on both monomers (decaying as ). In the small-*R*_*AB*_ limit, negative-transition terms attain the  dependence and the expression for the dispersion energy for a ground state molecule interacting with the excited state molecule presented in ref (47) becomes identical to that in eq 15 in the multipole expansion.

In this work, we study systems with a single lowest localized-exciton, for which repulsive dispersion forces are absent. A non-Casimir-Polder term pertains to μ < *I* = 1, ν > *J* = 0 [cf. the fourth term in eq 15] and it is negative. In the following, we compute the non-Casimir-Polder contributions directly from ground-state properties using a protocol introduced recently in ref (24).

## 3 Dispersion Energy from the Adiabatic Connection Theory

Let us consider a wave function description of an excited dimer and a supermolecular approach to computing the interaction energy. Assuming that the exciton is localized in the region of *A*, the simplest adequate wave function dissociates into multiconfigurational (MC) wave function  and a single-determinant ground state function 
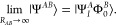
16The energy corresponding to such a wave function misses the intersubsystem correlation; in particular, the dispersion energy is not recovered.^48^ We now investigate how the dispersion energy emerges from the recently developed adiabatic connection theory for multiconfigurational wave functions.^21,49^

Begin by defining the correlation energy with respect to a reference state of interest Ψ as
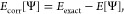
17where  is the reference energy corresponding to a model function Ψ. In the exact AC theory,^49^ the correlation energy is given by the expression involving integration along the adiabatic connection path
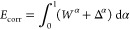
18The *W*^α^ integrand in the representation of the orthogonal orbitals {*p*, *q*, *r*, *s*, ... } reads
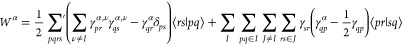
19where ⟨*pq*|*rs*⟩ denotes two-electron integrals and a prime in the first summation indicates that the correlation energy already accounted for by the wave function Ψ is excluded.^25^ All α-dependent quantities correspond to the adiabatic connection Hamiltonian 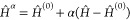
20
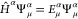
21By construction of , one of the eigenstates of  for the coupling constant α = 0 corresponds to the reference wave function
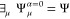
22that is, there exists a state μ in the manifold of states of the AC Hamiltonian which coincides with the chosen reference wave function at α = 0 (see also ref (49)). Transition density matrices γ^α,ν^, entering eq 19, defined as
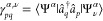
23involve the state  for which the index μ has been dropped, , and an arbitrary state . γ and γ^α^ denote one-electron reduced density matrices
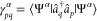
24
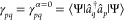
25

Creation and annihilation operators, , and , respectively, are in the representation of an arbitrary set of orthonormal spinorbitals.

We assume that the multiconfigurational reference wave function Ψ involves the partitioning of the orbitals *pqrs* into subsets of inactive (doubly occupied), active, and virtual (unoccupied) orbitals, and then the prime in eq 19 indicates those terms for which all orbitals *pqrs* are active are excluded. The Δ^α^ term in eq 18 originates from the mean field interactions between orbitals in different sets and depends on one-electron reduced density matrices (1-RDMs)^21,49^
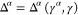
26

Consider a dimer in a nondegenerate state *A*_*I*_*B*_0_ defined by eq 16 and a contribution from the correlation energy to the pertinent supermolecular interaction energy
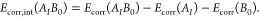
27To analyze the interaction energy at large intermonomer separation *R*_*AB*_, assume the basis set given as an union of orbitals *p*, *q*, *r*, *s*, ... and of *a*, *b*, *c*, *d*, ... completely localized in subsystems *A*_*I*_ and *B*_0_, respectively. Consequently, only the matrix elements , , and the two-electron integrals , , and  do not vanish. Begin with writing the AC Hamiltonian of a dimer as a sum of AC monomer Hamiltonians and the intermonomer interaction operator
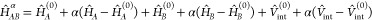
28Notice that out of the intermonomer interaction operator  components, only two-body (2b) operators
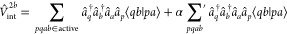
29are relevant for the dispersion energy. Under the assumption that the monomer *B* wave function is single-determinantal (orbitals *a*, *b* are not active), the first term vanishes and in the second term a prime symbol (indicating exclusion of terms for which all orbitals *pqrs* belong to the active set) can be skipped; that is, 
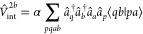
30which implies that at α = 0 there is no two-body interaction between monomers giving rise to the dispersion energy in the supermolecular interaction energy uncorrected for correlation.

Employing the AC correlation energy formula, eq 18, for both the dimer and the monomers in eq 27, and retaining only terms giving rise to the dispersion energy in the dissociation limit (notice that the Δ^α^ term depending only on one-particle reduced density matrices does not contribute), results in the AC dispersion interaction energy expression reading
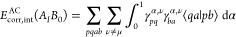
31where the notation ν ≠ μ means that terms connecting with the reference at α = 0 limit, cf. eqs 22–23, are excluded by construction. Notice that the AC dispersion energy in eq 31 includes terms higher than second-order in the interaction operator, so it is different from the second-order RS perturbation expression given in eq 15. In a special case when both monomers are in their ground states and they are described with single-determinantal wave functions, the AC dispersion energy is equivalent to the intermonomer correlation energy developed recently within the adiabatic connection symmetry-adapted perturbation theory.^50^

In the zeroth-order of the expansion with respect to the interaction operator, ν is a combined index describing states of the monomer A and B, ν_*A*_ and ν_*B*_, respectively. The products  differ from zero only for ν_*A*_ = *I* and ν_*B*_ = 0, but such terms are excluded from eq 31. The first nonzero term in  is obtained by employing the expression for first-order perturbation^39^ to a composite state  perturbed with 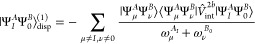
32in eq 31 leading to
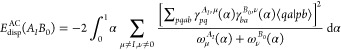
33Unlike the AC dispersion energy in eq 31, the expression shown in eq 33 is exact only in the second-order with respect to the interaction potential. It employs transition properties pertaining to isolated monomers in the AC formalism, that is, 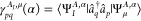
34
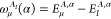
35where  and  are eigenfunctions and eigenvalues of the adiabatic connection Hamiltonian for the monomer A
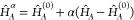
36namely
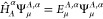
37(analogous definitions hold for *B*). Using the integral identity, eq 10, and density–density response functions of noninteracting monomers [cf. eq 3] described with the AC Hamiltonian  reading
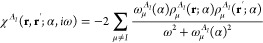
38(similarly for the monomer *B*), we are led to the formula for the second-order dispersion energy obtained in the adiabatic connection formalism:
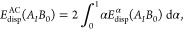
39where
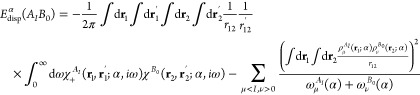
40and the  function is a positive-transition-energy component, using ν > *I* in eq 38, of the density–density response function of the noninteracting subsystem *A*.  is then the second-order dispersion energy as in eq 15, but corresponding to the α-dependent adiabatic connection Hamiltonian (cf. eq 28). At α = 0 the  dispersion energy pertains to uncorrelated, that is, described with the zeroth-order Hamiltonians, monomers. Retaining the description of monomers at the uncorrelated [α = 0 in eq 37] level, for each α in eq 40, leads to the uncoupled (UC) approximation for the dispersion energy
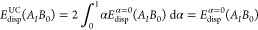
41used both in single reference^38,51^ and multireference^52^ molecular interaction theories. Integration along the adiabatic connection path, see eq 39, of the α-dependent energy  builds up electron correlation for each monomer and recovers the dispersion energy for the fully correlated interacting subsystems. The second term in eq 40 can be viewed as a non-Casimir-Polder term in the AC theory.

Notice that the AC dispersion energy presented in eq 40 is only asymptotically, that is, in the zero-overlap limit, equal to the expression derived from the perturbation theory, eq 15
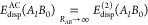
42

In this sense, the second-order perturbation theory and the adiabatic connection formalism are consistent in describing second-order dispersion energy in excited-state systems with localized exciton if the wave function describing interacting monomers satisfies the condition given in eq 16. Notice that when both monomers are described with MC wave functions, that is, active orbitals are localized on both monomers, the AC-based dispersion energy differs from its second-order counterpart even asymptotically, since it accounts only for the residual dispersion energy.^48^

Both AC and the second-order perturbation theories for describing correlation interaction energy form a ground for approximate methods dedicated to molecular interactions, as shown below.

## 4 Correcting Supermolecular Interaction Energy for the Long-Range Correlation Energy

Investigation of intermolecular interactions in excited-state systems with wave function theory demands size-consistency of the assumed multiconfigurational wave function model. In the case of variational MCSCF methods, CASSCF in particular, the wave function is constructed as an antisymmetrized product of the Slater determinant, Φ, formed from the inactive orbitals, and the MC function, Ψ_act_, constructed from the active orbitals
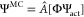
43(the operator  is an antisymmetrizer with the normalization factor). If such an ansatz is used for a dimer, the size-consistency condition implies that for each component Φ and Ψ_act_ the following conditions hold
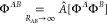
44
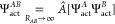
45where the monomers’ wave functions are
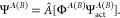
46Such a group-product size-consistency condition implies the energy size-consistency.

As discussed in ref (48), the supermolecular interaction energy expression obtained by employing the multiconfigurational wave function captures only a marginal portion of the dispersion energy. If only one monomer is described with a MC wave function, then the dispersion energy is entirely missing from the supermolecular interaction energy. Clearly, accounting for the long-range correlation is crucial when applying multiconfigurational models to noncovalent interactions.

### CAS+DISP

One of the possible solutions is a direct addition of the missing dispersion energy, as proposed in ref (48). The CAS+DISP method^48^ employed in this work consists of adding perturbation-theory-based dispersion energy, eq 2, together with the dispersion-exchange component to the CAS interaction energy
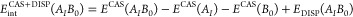
47where
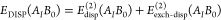
48Response properties of the monomers entering the computation of both the  and  terms follow from solutions of ERPA^22,53^ equations, as described in detail in our earlier works.^23,52^

### AC0-CAS

The adiabatic connection approach, see eq 18, allows one, in principle, to recover the correlation energy for CASSCF wave functions exactly.^49^ Approximate multiconfigurational AC methods assume fixing the electron density along the AC path.^21,25,49,54−56^ These approaches employ ERPA equations^22,53^ for the computation of the linear response for the AC Hamiltonian. As it has been shown in ref (57), ERPA is size-consistent, which implies that the AC methods combined with CAS are suitable for studying the molecular interactions.

The most efficient variant of ERPA-based adiabatic connection methods, referred to as AC0, is based on the linear expansion of the AC integrand *W*^α^, see eq 19, at α = 0
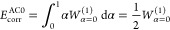
49By comparing the explicit form of the  expression given in eq 46 in ref (25) with the formula for the dispersion energy in the uncoupled approximation [see eq 26 in ref (52)], it follows immediately that the dispersion energy predicted by the AC0 method is described at the uncoupled level of theory, cf., eq 41
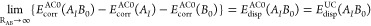
50In the AC0-CAS method, the interaction energy is obtained by computing AC0 correlation energy for a dimer and monomers from pertinent CAS wave functions (in fact, only 1- and 2-RDMs from CAS are needed); that is, 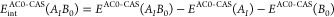
51where
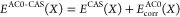
52

### lrAC0-CAS

The CAS interaction energy in the CAS+DISP approach is corrected for the dynamic correlation only in the long-range of electron–electron interaction and it inevitably misses the short-range electron correlation. Although the AC0 correlation correction covers the entire range of electron correlation, it involves random phase approximation. AC0-CAS is thus expected to give a less accurate description of the short-range correlation effects compared to density functional approximations.^58^ To exploit this advantage of DFT over *ab initio* methods, we have recently proposed to constrain the range of the AC0 description by deriving its long-range variant, and combining it with the short-range PBE exchange-correlation functional .^59^ The resulting lrAC0-CAS energy expression^27^
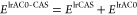
53where
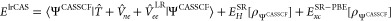
54is employed in a post-CAS fashion; that is, the CASSCF wave function Ψ^CASSCF^ and the electron density  follow from CAS calculations with the Hamiltonian including the full-range electron interaction operator. Range-separation of the electron correlation into the long-range (LR) and short-range (SR) parts is governed by a parameter μ, such that the AC0-CAS is a limiting case of lrAC0-CAS if μ → *∞*.^27^ In our calculations, μ has been set to 0.5 bohr^–1^.

### SAPT(MC)

One of the methods suitable for studying molecular interactions in the excited state is the recently developed multiconfigurational symmetry adapted perturbation theory, SAPT(MC).^24^ This approach has two advantages over the supermolecular method. First, SAPT requires only monomer properties, so there is no need to compute a dimer wave function. For MC wave functions, the latter may be problematic if size-consistency is to be preserved. The second appealing feature of SAPT is that the interaction energy is given as a sum of physically meaningful components which provide insight into the character of the interaction.

The SAPT(MC) formalism is dedicated to multireference systems. It can be applied with any *ab initio* model that gives access to 1- and 2-RDMs of monomers. The method predicts the interaction energy up to the second-order terms in the interaction operator
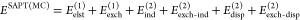
55Both first- and second-order exchange terms employ the *S*^2^ approximation,^60^ while the density response functions that enter the second-order terms are described at the ERPA level of theory. For a general expression for the dispersion energy used in SAPT see eq 2.

SAPT(MC) combined with CASSCF description of the monomers leads to the method called SAPT(CAS). To improve SAPT(CAS) accuracy for systems with large polarization effects, one needs to account for higher-order induction terms. For monomers in their ground states, these terms can be approximated at the Hartree–Fock (HF) level of theory^61,62^ and represented as the δ_HF_ correction
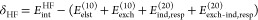
56where  corresponds to the supermolecular HF interaction energy and the double (*ij*) superscript refers to the *i*th order in the intermolecular perturbation and the *j*th order in the intramolecular perturbation. When considering monomers in their excited states, there is no trivial way to obtain the equivalent of the δ_HF_ correction. To circumvent this problem, we assume that a shift in higher-than-second-order induction terms following generation of an exciton is proportional to a corresponding change of the second-order induction and propose a δ_CAS_ analogue of the δ_HF_ term calculated as
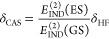
57where  and ES/GS denote dimers in excited and ground states, respectively. We have applied the δ_CAS_ correction in all SAPT(CAS) calculations for interactions involving excited states.

### Nonlocal Density Correlation Functional: reVV10

The approximate methods discussed thus far rely on the dispersion energy computed from one- and two-electron reduced density matrices of monomers. In DFT, the dispersion energy should be attainable using only electron densities or Kohn–Sham orbitals and orbital energies of monomers. The latter quantities are used in SAPT(DFT),^63,64^ which provides accurate predictions for the second-order dispersion energy. The method is not applicable, however, to singlet excited systems, studied in this work. A viable alternative within the Kohn–Sham framework are nonlocal correlation energy density functionals, capable of describing long-range correlation.^65,66^ They were designed to cure the deficiencies of the local and semilocal functionals in describing van der Waals systems. In particular, the VV10 correlation energy functional^28^ introduced by Vydrov and van Voorhis has gained popularity in applications to noncovalently bound molecular complexes due to its simple form, afforded accuracy, and the possibility to couple it with different density functional approximations. The excellent performance of the VV10 model combined with various exchange-correlation functionals (see e.g., refs (67 and 68)) has motivated us to adapt it to the dispersion-free CASSCF method in order to describe molecular interactions. This required reparameterizing VV10, so that the functional would capture only the missing part of the long-range correlation in the supermolecular CAS, that is, the dispersion energy. A similar idea to design a functional reproducing the second-order dispersion has recently been explored by Shahbaz and Szalewicz.^69^ Their functional achieves good accuracy, but its applicability is limited to systems with a clear separation into weakly interacting monomers. Employment of the VV10 correlation functional for describing dispersion energy is free of this limitation.

The VV10 expression for the nonlocal correlation energy written in atomic units reads
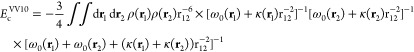
58(notice that a constraint of vanishing VV10 for uniform densities is not imposed), where the local excitation frequency ω_0_(**r**) is defined as
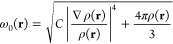
59An adjustable parameter *C* governs the behavior of the integrand in eq 58 in the r_12_ → *∞* limit. The κ(**r**) function
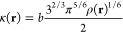
60playing a dominant role in the short-range interelectron distance regions, includes a parameter *b*. The aim of combining VV10 with CASSCF is to correct for the missing dispersion energy in the latter. For this purpose the parameters *C* and *b* must be tuned, so that the VV10 nonlocal correlation interaction energy, 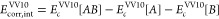
61matches the sum of the second-order dispersion and exchange-dispersion. In this work, we reparameterized VV10 to minimize the error of counterpoise-corrected VV10 correlation interaction energy, see eq 61, for the training set consisting of argon, water, and ethanol dimers (cf., Supporting Information for details). The SAPT(DFT) dispersion and exchange-dispersion energies taken from ref (69) are used as benchmarks in training the functional. We found the optimal values of *C* = 0.013 and *b* = 2.84 to be compared with *C* = 0.0093 and *b* = 5.9 obtained in ref (28) for VV10 combined with the rPW86^70^ exchange and PBE^71^ correlation functionals. The smaller *b* value in the dispersion-optimized functional than that in the original work means that correlation interaction energies following from reparameterized VV10 are more binding. This is understandable, since VV10 combined with rPW86-PBE must account for only a fraction of the long-range correlation energy when densities of the interacting fragments overlap, the rest is recovered by the exchange-correlation density functional.

The optimized VV10 has been tested on a set consisting of 8 molecular dimers (Ar–HF, nitromethane, methylformate, benzene–methane, benzene–water, imidazole, nitrobenzene, and ethylenedinitramine dimers) and 60 data points, achieving mean absolute percentage error and mean error of 14% and −0.13 kcal·mol^–1^, respectively. The details of the calculations are presented in the Supporting Information. The reparameterized VV10 correlation functional will be referred to as reVV10, and the CASSCF interaction energy corrected for the long-range correlation obtained as shown in eq 61 will be denoted as CAS-reVV10.

On a final note, since reVV10 is parametrized explicitly for the dispersion energy, it can be combined with any “dispersion-free” model, such as the supermolecular Hartree–Fock interaction energy or dispersionless DFT approaches.^72,73^

## 5 Noncovalent Interactions in π → π* and n → π* Excited Systems

### 5.1 Computational Details

**Figure 1:**
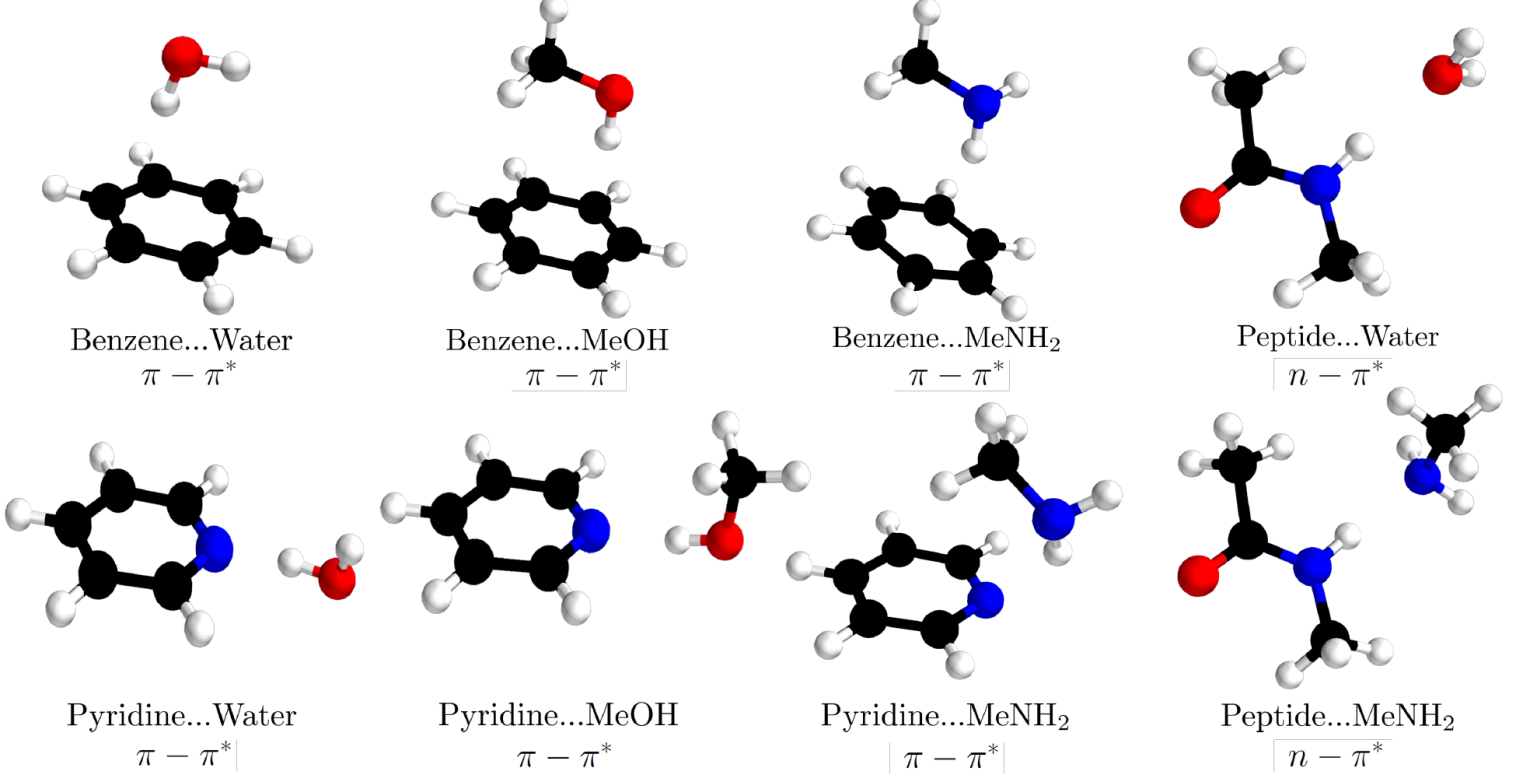
Structures of eight complexes in their ground state geometries. Interaction energies in the lowest π–π* (benzene and pyridine complexes) and n−π* (peptide complexes) excited states are studied in this work.

For this study, we selected eight complexes from the S66 benchmark data set^74,75^ of Hobza and co-workers: benzene–water, benzene–MeOH, benzene–MeNH_2_, pyridine–water, pyridine–MeOH, pyridine–MeNH_2_, peptide–water, peptide–MeNH_2_ (peptide corresponds to *N*-methyl-acetamide), shown in Figure 1. Both ground- and excited-state calculations employed the original S66 geometries optimized for the ground state at the MP2/cc-pVTZ level of theory. The Boys–Bernardi counterpoise correction was applied to eliminate the basis set superposition error (BSSE).^76^ The excitons in excited states calculations were localized on benzene (π → π*), pyridine (π → π*), and peptide (n → π*) molecules. As a benchmark for the interaction energy in ground state dimers we adopted the CCSD(T) results extrapolated to the complete basis set limit (CBS) from ref (74). Reference values of the interaction energy in complexes involving excited states were taken from ref (20). They were obtained by combining the CCSD(T)/CBS description of the ground state with excitation energies calculated at the EOM-CCSD^77^ level of theory using the 6-31++G(d,p) basis set.^78−80^

All CASSCF computations employed the aug-cc-pVTZ basis set^81^ and were performed in the Molpro^82^ program. The MP2 orbitals were used as a starting guess for CASSCF. The active space composition was identical for ground- and excited-states. Benzene active space involved six active electrons on six orbitals, the three π bonding and the three π* antibonding MOs, labeled as CAS(6,6).^83^ The active space for pyridine included the three π bonding and three π* antibonding orbitals of the pyridine ring along with one nitrogen’s lone pair, CAS(8,7).^84^*N*-methyl-acetamide (peptide) active space was composed of σ_CO_, π_CO_,  and  orbitals, and two lone pair orbitals located on oxygen *n*_O_.^85^ To obtain the ground- and excited-state wave functions of both the dimer and one of the monomers, we carried out two-state state-averaged CAS computations.

Note that orbital rotations are usually required to maintain size-consistency in CASSCF, even when MP2 or CI natural orbitals are employed as the initial guess. To ensure size-consistency, we first converged a CASSCF dimer wave function with monomers separated by 100 Å, confirming that the supermolecular interaction energy vanishes. The resulting orbitals served as the initial guess for equilibrium geometry dimer calculations.

All CAS+DISP, AC0-CAS, lrAC0-CAS, CAS+reVV10, and SAPT(CAS) calculations were performed in the GammCor program.^86^ The required electron integrals, 1- and 2-RDMs for CASSCF wave functions were obtained in the locally modified Molpro package. The latter program was also used to carry out CASPT2^87^ calculations.

As previously discussed, see eq 15, the dispersion energy for excited-state systems includes non-Casimir-Polder terms resulting from negative transitions. For the lowest excited states considered there is one negative excitation 1 → 0. Consequently, the sum of the aforementioned terms reads [set *I* = 1 and *J* = 0 in eq 6]
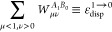
62The procedure proposed in ref (24), implemented in GammCor, was used to compute the non-Casimir-Polder  term for each dimer.

### 5.2 Insights from a SAPT(CAS) Analysis

Let us first examine the SAPT(CAS) results and analyze the changes in interaction energy components induced by vertical excitations. Beginning with the complexes of benzene interacting with H_2_O, MeOH, and MeNH_2_ molecules, we note that in the ground state all complexes are bound due to an X–H···π interaction, which is of a mixed electrostatic and dispersion character. The  to  ratio computed for the ground state amounts to 1.1, 1.6, and 2.2 for benzene–H_2_O, −MeOH, and −MeNH_2_ complexes, respectively (see Table S2 in the Supporting Information). Evidently, the methylamine complex can be considered dispersion-dominated, in agreement with data reported in ref (74). When the benzene-localized exciton is generated, the dispersion to electrostatic energy ratios increase to 1.6, 2.2, 2.8 (see Table 1) and the interaction is more dispersion-driven than in ground states. At the same time, inspection of the SAPT(CAS) results reported in Table 1 shows that the X–H···π interaction is destabilized by the π–π* excitation on benzene in all three complexes (in agreement with the benchmark values, see below). Analysis of the shifts in values of the SAPT(CAS) energy components triggered by the excitation provides an insight into the mechanisms behind the destabilization. Namely, electron density redistribution from π to π* orbitals decreases the electrostatic attraction by 0.88, 0.98, and 0.54 kcal·mol^–1^. This drop is paralleled by a lowered exchange repulsion, but the latter effect is not sufficient to compensate for the decrease in electrostatics. Another source of destabilization of the benzene complexes in the excited state is the reduction of the dispersion interaction by 0.17, 0.24, and 0.22 kcal·mol^–1^. This may seem counterintuitive, as it is a common understanding that the dispersion attraction should increase in excited states due to the increased static polarizability, see the discussion in ref (20). One concludes that in the π–π* state the X–H···π interaction is weakened compared to the ground state. Although it remains dominated by both electrostatic and dispersion energy contributions, the electrostatic component is substantially smaller than in ground states.

**Table 1:** Upper part of the Table Presents Interaction Energy Components of SAPT(CAS), their Sums (*E*_int_^SAPT^), and non-Casimir-Polder Terms (ε_disp_^1→0^) for Excited State Complexes. Differences of SAPT(CAS) Energies between Excited (e.s.) and Ground States (g.s.), Δ*E*_*x*_ = *E*_*x*_(e.s.) – *E*_*x*_(g.s.), are Shown in the Lower Part of the Table. All Values Are Reported in kcal·mol^–1^

	*E*_elst_^(1)^	*E*_exch_^(1)^	*E*_ind_^(2)^	*E*_exch-ind_^(2)^	*E*_disp_^(2)^	*E*_exch-disp_^(2)^	*E*_int_^SAPT^	ε_disp_^1→0^
benzene–water	–1.85	2.82	–1.23	0.65	–2.88	0.33	–2.16	–0.04
benzene–MeOH	–2.10	4.07	–1.57	0.96	–4.63	0.52	–2.76	–0.06
benzene–MeNH_2_	–1.68	3.73	–1.12	0.88	–4.62	0.54	–2.28	–0.02
pyridine–water	–11.23	10.66	–5.17	2.96	–4.05	0.84	–5.99	–0.07
pyridine–MeOH	–11.79	11.79	–5.92	3.53	–4.95	0.99	–6.37	–0.08
pyridine–MeNH_2_	–3.89	5.46	–1.79	1.30	–5.01	0.66	–3.27	–0.08
peptide–water	–5.99	5.33	–1.95	1.01	–2.93	0.46	–4.09	0.00
peptide–MeNH_2_	–9.84	10.91	–5.04	3.35	–5.78	1.10	–5.30	0.00

	Δ*E*_elst_^(1)^	Δ*E*_exch_^(1)^	Δ*E*_ind_^(2)^	Δ*E*_exch-ind_^(2)^	Δ*E*_disp_^(2)^	Δ*E*_exch-disp_^(2)^	Δ*E*_int_^SAPT^	
benzene–water	0.88	–0.35	0.11	–0.05	0.17	–0.05	0.72	
benzene–MeOH	0.98	–0.45	0.15	–0.08	0.24	–0.07	0.77	
benzene–MeNH_2_	0.54	–0.25	0.08	–0.03	0.22	–0.05	0.50	
pyridine–water	–0.04	0.02	0.02	0.01	0.02	0.00	0.03	
pyridine–MeOH	–0.03	0.02	0.02	0.01	0.04	0.00	0.04	
pyridine–MeNH_2_	0.17	–0.15	0.04	–0.04	0.15	–0.03	0.14	
peptide–water	0.71	–0.03	0.12	–0.03	–0.01	0.01	0.77	
peptide–MeNH_2_	0.71	0.05	–0.12	0.32	–0.10	0.05	0.91	

In hydrogen-bonded peptide–water and peptide–methylamine dimers, the interaction energy also decreases upon the n−π* excitation. The SAPT(CAS) explanation is the same as in the case of benzene complexes, namely the hydrogen bond formed by peptide is weakened as a result of the decreased electrostatic attraction. The latter lowers by 0.71 kcal·mol^–1^ for both complexes (see Table 1). This is expected, since the n−π* excitation reduces the electron density on the nitrogen atom of the peptide, which serves as a hydrogen-bond acceptor. The change in the dispersion energy is negligible in the complex with water. For the peptide–methylamine dimer the dispersion increase is visible, yet it is only a minor effect of 0.10 kcal·mol^–1^ (below 2% of the ). Therefore, SAPT identifies destabilization of the hydrogen bonded systems upon the vertical n−π* excitation as a mainly electrostatic effect. This remains in agreement with the energy decomposition analysis study of Head-Gordon and co-workers.^32^

The last group of excited complexes investigated in this work comprises pyridine interacting with water, methanol, and methylamine molecules. The first two complexes are hydrogen-bonded,^74^ which is confirmed by the low dispersion to electrostatic energies ratio, amounting to 0.4 in the ground state (see Table S2 in the Supporting Information). For the pyridine–methylamine dimer the magnitude of the dispersion energy is of the order of the electrostatic term (compare −5.17 vs −4.06 kcal·mol^–1^), and the character of the interaction is therefore mixed. The considered π–π* interaction does not involve nitrogen lone pair electrons of pyridine, so that H-bonded complexes are practically unaffected by the excitation. Indeed, differences in SAPT(CAS) energy components between ground and excited states for H-bonded pyridine–water and pyridine–methanol complexes do not exceed 0.04 kcal·mol^–1^. For the methylamine complex one observes a positive shift in the interaction energy by 0.14 kcal·mol^–1^. It results from decreased electrostatic and dispersion attractive interactions. The magnitudes of these two energy components are lowered by, respectively, 0.17 and 0.15 kcal·mol^–1^. These changes are not compensated by a negative shift of the exchange energy, which in the excited state is by 0.15 kcal·mol^–1^ lower than in the ground state.

### 5.3 Assessment of Correlation-Energy-Corrected CASSCF-Based Methods

In Table 2 we show contributions to the interaction energies in excited-state dimers from the intermonomer electron correlation computed as *E*_corr_(*AB*) – *E*_corr_(*A*) – *E*_corr_(*B*), where *E*_corr_ denotes AC0, lrAC0, and reVV10. For comparison, we have also included the dispersion energy obtained within the local response dispersion model, LRD, proposed by Nakai et al. for ground and excited states.^20,88^ The LRD energy values reported in Table 2 have been taken from ref (20). The correlation interaction energies presented in Table 2 are confronted with the dispersion energy *E*_DISP_ combining the second-order dispersion and the exchange-dispersion terms, cf. eq 48. Since in our calculations CAS interaction energy misses entirely the dispersion energy contribution,^48^ the prime role of the correlation interaction energy added to CAS is to compensate for the lack of the dispersion energy not only in the long intermolecular distance, but also when electron densities of interacting fragments overlap.

**Table 2:** AC0, lrAC0, reVV10, and LRD Correlation Energy Contributions to Interaction Energies for π–π* (Benzene and Pyridine Complexes) and n−π* (Peptide Complexes) Excited State Systems Confronted with the Sum of the Dispersion and Exchange-Dispersion Energy *E*_DISP_ (see eq 48). Values Are Reported in kcal·mol^–1^

	*E*_DISP_	AC0	lrAC0	reVV10	LRD
benzene–water	–2.55	–2.50	–2.93	–3.04	–1.07
benzene–MeOH	–4.11	–3.58	–4.70	–4.99	–1.84
benzene–MeNH_2_	–4.08	–3.91	–4.51	–4.97	–1.95
pyridine–water	–3.21	–2.41	–3.14	–2.16	–0.56
pyridine–MeOH	–3.96	–2.71	–3.90	–2.97	–0.92
pyridine–MeNH_2_	–4.34	–4.04	–4.72	–4.85	–1.71
peptide–water	–2.47	–2.00	–2.29	–2.07	–0.53
peptide–MeNH_2_	–4.69	–4.10	–4.74	–4.15	–1.30

It is evident from Table 2, that the AC0 correlation interaction energies are systematically smaller in magnitude than *E*_DISP_. Recall that AC0 accounts for the uncoupled dispersion energy only in the asymptotic regime, eq 50. Clearly, this is not fulfilled when monomer densities overlap (see also Table S3 in the Supporting Information for comparison with uncoupled dispersion terms).

Employing the range-separation of electron interaction operator and constraining the AC0 correction to the long-range, as it is done in the lrAC0 approach, removes the systematic underestimation and relative deviations from *E*_DISP_ fall in the 1–15% range.

The VV10 functional, reparameterized in this work to reproduce the pure dispersion energy (*E*_DISP_) in supermolecular calculations for ground states, deviates from the true *E*_DISP_ by more than 0.5 kcal·mol^–1^ (11–33% deviations in terms of relative errors). The correlation energy from the LRD model of Nakai et al.^19,88^ does not match the dispersion energy, the LRD energy being 2–3 times smaller than *E*_DISP_. This implies that LRD recovers only a part of the long-range correlation (dispersion) interaction energy and the remaining (middle-range) correlation is captured by the correlation energy functional with which LRD is paired. A good level of accuracy of LRD combined with the long-range corrected LC-BOP functional and the pertinent TD-DFT excitation energies, see Table 4, is achieved due to the tuning of both the range-separation parameter in the exchange functional and parameters in the LRD correction. Notice that the recommended values of the range-separation parameter for the LC-BOP functional combined with LRD are different for ground and the excited states (0.47 and 0.33 au, respectively).^20^

**Figure 2:**
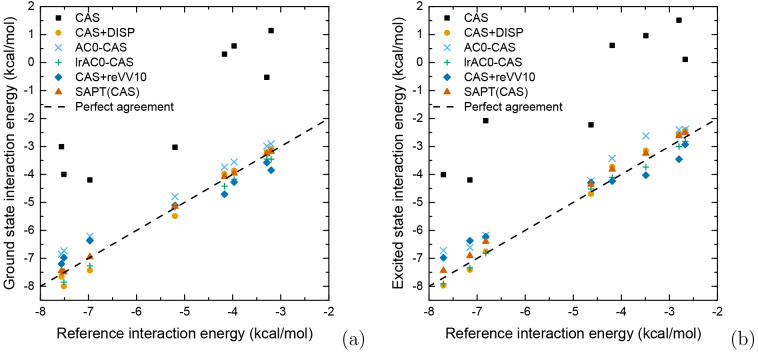
Correlation plots for interaction energies for complexes in ground (panel a) and excited (panel b) states.

Tables 3 and 4 and Figure 2 present the interaction energies for ground and excited states obtained with uncorrected CASSCF, correlation-energy-corrected CASSCF methods, and SAPT(CAS) supplemented with δ_HF_ (ground states) and δ_CAS_ terms (excited states). For comparison, LC-BOP-LRD interaction energies from ref (20) have been included.

**Table 3:** Ground State Interaction Energies in kcal·mol^–1^^a^

	CAS	CAS+DISP	AC0-CAS	lrAC0-CAS	CASPT2	CAS-reVV10	SAPT	LC-BOP+LRD	ref.
benzene–water	–0.53	–3.20	–2.99	–3.45	–3.13	–3.58	–3.27	–3.33	–3.29
benzene–MeOH	0.30	–3.98	–3.74	–4.42	–4.14	–4.71	–4.08	–3.91	–4.17
benzene–MeNH_2_	1.14	–3.10	–2.91	–3.46	–3.29	–3.85	–3.18	–2.92	–3.20
pyridine–water	–4.20	–7.43	–6.21	–7.27	–5.44	–6.37	–6.95	–7.17	–6.97
pyridine–MeOH	–4.00	–8.00	–6.73	–7.85	–7.22	–6.98	–7.49	–7.50	–7.51
pyridine–MeNH_2_	0.59	–3.87	–3.56	–4.17	–4.02	–4.28	–3.96	–3.55	–3.97
peptide–water	–3.03	–5.49	–4.79	–5.18	–4.94	–5.11	–5.15	–5.09	–5.20
peptide–MeNH_2_	–3.01	–7.65	–6.85	–7.50	–7.37	–7.20	–7.45	–7.16	–7.56
MUE	3.64	0.23	0.51	0.20	0.33	0.42	0.04	0.22	
MA%E	78.75	4.10	9.67	4.25	5.37	9.03	0.83	4.65	

a CCSD(T)/CBS results from ref (74) are given as reference in the last column. The SAPT acronym refers to SAPT(CAS) results including the δ_HF_ correction [see eq 56]. Mean unsigned errors (MUE) and mean absolute percentage errors (MA%E) are computed with respect to the reference.

**Table 4:** Interaction Energies in kcal·mol^–1^ for π–π* (Benzene and Pyridine Complexes) and n−π* (Peptide Complexes) Excited States^a^

	CAS	CAS+DISP	AC0-CAS	lrAC0-CAS	CASPT2	CAS-reVV10	SAPT	LC-BOP+LRD	ref
benzene–water	0.11	–2.43	–2.39	–2.82	–3.12	–2.93	–2.51	–2.88	–2.67
benzene–MeOH	0.96	–3.15	–2.62	–3.74	–3.42	–4.03	–3.25	–3.55	–3.49
benzene–MeNH_2_	1.51	–2.57	–2.40	–3.00	–3.24	–3.46	–2.62	–2.74	–2.80
pyridine–water	–4.20	–7.41	–6.61	–7.34	–7.90	–6.37	–6.91	–7.96	–7.15
pyridine–MeOH	–4.01	–7.97	–6.72	–7.91	–7.21	–6.98	–7.44	–8.37	–7.70
pyridine–MeNH_2_	0.61	–3.73	–3.43	–4.11	–3.96	–4.24	–3.82	–4.06	–4.19
peptide–water	–2.23	–4.70	–4.23	–4.52	–4.92	–4.29	–4.36	–4.81	–4.63
peptide–MeNH_2_	–2.08	–6.76	–6.18	–6.82	–7.28	–6.23	–6.40	–6.97	–6.82
MUE	3.77	0.24	0.61	0.15	0.40	0.49	0.23	0.28	-
MA%E	88.83	5.93	13.27	3.70	8.74	10.77	4.85	5.12	-

a The SAPT acronym refers to SAPT(CAS) results including the δ_CAS_ correction [see eq 57]. The Est. EOM-CCSD(T) values from ref (20) are given as reference in the last column. Mean unsigned errors (MUE) and mean absolute percentage errors (MA%E) are computed with respect to the reference.

The CASSCF interaction energies are, as expected, severely underestimated for both ground and excited states, as a consequence of CASSCF missing entirely the dispersion interaction.^48^ This deficiency is most striking in the case of dispersion-dominated excited benzene complexes (Table 1), which are predicted as unbound by CASSCF. A significant improvement is achieved for all systems when the dispersion correction is added to supermolecular CASSCF energies, as shown in eq 47. The mean unsigned error (MUE) of 0.2 kcal·mol^–1^ is achieved by the CAS+DISP methods before and after generation of the exciton in the considered systems. This translates into mean absolute percentage errors (MA%E) of 4% and 6% for ground- and excited states, respectively. Since supermolecular CASSCF misses the majority of intramonomer correlation effects, the good performance of CAS+DISP should be, to some extent, attributed to error cancellation.

Using the approximate adiabatic connection correlation correction for CAS, as it is done in the AC0-CAS method, leads to interaction energies that are systematically underestimated. The MUE and MA%E values corresponding to AC0-CAS amount to 0.61 kcal·mol^–1^ and 13%, respectively, for the excited complexes (Table 3). This places the AC0-CAS method as the least accurate (except for uncorrected CASSCF) of all the considered approximations.

Out of the two sources of electron interaction inaccuracies obtained with AC0-CAS, the insufficient electron correlation at middle-ranges due to the underlying extended RPA and description of the dispersion energy at the uncoupled level, the former seems to be of prime importance. This is corroborated by lrAC0-CAS results, for which the short- and middle-range correlation is described efficiently within DFT. As shown in Tables 3 and 4, lrAC0-CAS yields interaction energies of an excellent accuracy with the mean errors of 0.20 kcal·mol^–1^ for ground states and 0.15 kcal·mol^–1^ for localized-exciton systems (corresponding to a MA%E value of 4% in both cases). The spread of errors is small, and the maximum deviation from the reference amounts to 0.25 kcal·mol^–1^. In fact, lrAC0-CAS offers the highest accuracy of all the considered approaches. The excellent performance of lrAC0-CAS is achieved by a balanced description of the electron correlation at short, middle, and long-range regimes by a density-functional and the adiabatic-connection correlation. This feature is missing in the CASPT2 method, where correlation energy results from the second-order perturbation correction. The averaged CASPT2 error for excited-state complexes is rather large, amounting to 0.40 kcal·mol^–1^, and the method tends to overestimate the interaction energy of the excited systems by as much as 0.75 kcal·mol^–1^ for the pyridine–water complex. Ground state (closed shell) interaction energies predicted by CASPT2 are mostly underestimated (Table 3) and the MUE of 0.33 kcal·mol^–1^ is lower than after excitation.

It is worthwhile to compare the performance of methods that employ density functionals to recover the long-range correlation, that is, CAS-reVV10 and LC-BOP+LRD. In spite of the fact that both approaches rely on parameters obtained for ground states, they retain a similar level of accuracy for excited-state complexes. The CAS-reVV10 method deviates from the CC benchmark by 9% and 11% for ground states and excited states, respectively. Taking into consideration that CAS-reVV10 is of the lowest computational cost of all the considered approximations, the MUE of 0.49 kcal·mol^–1^ is considered acceptable. LC-BOP+LRD has an average error of half of that of CAS-reVV10. Notice, however, that the computational cost of the LRD correction for excited states is higher than that for reVV10, as it requires solving the TD-DFT equations. Both methods miss the contribution of the non-Casimir-Polder terms to dispersion, which will be a problem when these terms play a decisive role.^24^

SAPT(CAS) supplemented with the δ_CAS_ correction affords a similar level of accuracy as the CAS+DISP model for excited-state systems, with MA%E values amounting to 6% in both cases. Since the considered systems are either H-bonded or of a mixed character, polarization effects cannot be neglected:^89^ for excited states the δ_CAS_ terms reduce the mean unsigned error of SAPT(CAS) from 0.90 kcal·mol^–1^ to 0.23 kcal·mol^–1^. Further reduction of MUE to 0.18 kcal·mol^–1^ follows after adding the non-Casimir-Polder terms shown in Table 1. The excellent results obtained for ground-state complexes (MA%E of 1%) should be attributed to a systematic error cancellation between attractive and repulsive terms, which had also been observed in a previous SAPT(CAS) study.^24^

## 6 Summary and Conclusions

This work has addressed the problem of intermolecular interaction energy calculations in molecular complexes with localized excitons. Our aim has been to provide a relevant theoretical description of the dispersion energy. We have derived a generalized Casimir-Polder formula involving terms that result from negative electron transitions and are specific for excited states. While these terms are negative for systems with single excitons, they become positive for multiple excitons localized on both monomers.

Apart from the perturbation-based expression, we have derived the AC formula for the dispersion energy and its lowest-order-expansion with respect to the interaction potential. This approach recovers the dispersion interaction through integration of the coupling parameter-dependent dispersion energy corresponding to the AC Hamiltonian.

A numerical demonstration was carried out for a few dimers from the S66 data set. The excitons in the studied systems were localized on benzene, pyridine, and peptide molecules forming complexes with water, methanol, and methylamine.

To examine interactions driven by the creation of a local exciton, we have performed a SAPT(CAS) analysis. In linear hydrogen-bonded peptide complexes the decrease in the interaction strength upon n−π* excitation was attributed to the decline in the electrostatic attraction. In on-top X–H···π structures of benzene and pyridine dimers the diminished electrostatic energy upon π–π* excitation remains the dominant effect, yet substantially compensated by first-order exchange. Importantly, in on-top complexes the change in second-order terms—dispersion and induction—is smaller compared to that in first-order, but cannot be neglected. When corrected for the induction energy terms beyond the second order, SAPT(CAS) yielded mean errors of 0.2 kcal·mol^–1^ (5% in terms of mean relative percentage errors).

SAPT(CAS) has also been employed for the evaluation of the extended Casimir-Polder formula proposed in this work. The non-Casimir-Polder terms computed for the lowest valence excitations did not exceed −0.1 kcal·mol^–1^. In general, however, non-Casimir-Polder contributions can be larger, see, for example, ref (24). For such cases, methods neglecting the negative excitations in dispersion for excited states are bound to fail.

Next to perturbation-based and AC treatments of the dispersion energy, we adapted a VV10 nonlocal correlation density functional to the dispersionless CAS wave function. The parameters in the functional have been trained on benchmark dispersion energies. CAS-reVV10 interaction energies yield errors averaging around 0.5 kcal·mol^–1^ for excited state complexes. Taking into account the low computational cost of the reVV10 correction, CAS-reVV10 may prove useful for large systems with localized excitons, for which non-Casimir-Polder terms are negligible.

We examined approximate methods which correct CASSCF for the missing electron correlation. A direct addition of the second-order dispersion energy to the CASSCF interaction energy (CAS+DISP) proved a viable approach, with the averaged error of 0.24 kcal·mol^–1^. As expected, AC0 correlation energy, which employs the extended random phase approximation, leads to a systematic underbinding. Greatly improved results are obtained when a density correlation functional is used for short-ranged electron correlation, and the AC0 is limited to long-range. This strategy, implemented in the lrAC0-CAS method, has reduced the mean error of AC0-CAS from 0.61 kcal·mol^–1^ to 0.15 kcal·mol^–1^ on the set of excited complexes. The range-separated lrAC0-CAS model ranks as the most accurate approach for studying interaction energies in excited state complexes.

## Supporting Information Available

Details of the VV10 correlation functional reparameterization; SAPT(CAS) energies for complexes in ground states; AC0 correlation energy contribution to the interaction energy compared with the uncoupled and coupled dispersion energy for ground and excited states (PDF)

## References

[ref1] Bredas J.-L.; Beljonne D.; Coropceanu V.; Cornil J. Charge-Transfer and Energy-Transfer Processes in π-Conjugated Oligomers and Polymers: A Molecular Picture. Chem. Rev. 2004, 104, 4971–5004. 10.1021/cr040084k. 15535639

[ref2] Scholes G. D. Long-Range Resonance Energy Transfer in Molecular Systems. Annu. Rev. Phys. Chem. 2003, 54, 57–87. 10.1146/annurev.physchem.54.011002.103746. 12471171

[ref3] Mei J.; Leung N. L. C.; Kwok R. T. K.; Lam J. W. Y.; Tang B. Z. Aggregation-Induced Emission: Together We Shine, United We Soar. Chem. Rev. 2015, 115, 11718–11940. 10.1021/acs.chemrev.5b00263. 26492387

[ref4] Wang W.; Zhang Y.; Jin W. J. Halogen bonding in room-temperature phosphorescent materials. Coord. Chem. Rev. 2020, 404, 213107 10.1016/j.ccr.2019.213107.

[ref5] Zhu W.; Zheng R.; Zhen Y.; Yu Z.; Dong H.; Fu H.; Shi Q.; Hu W. Rational Design of Charge-Transfer Interactions in Halogen-Bonded Co-crystals toward Versatile Solid-State Optoelectronics. J. Am. Chem. Soc. 2015, 137, 11038–11046. 10.1021/jacs.5b05586. 26226301

[ref6] Fabrizio A.; Corminboeuf C. How do London Dispersion Interactions Impact the Photochemical Processes of Molecular Switches?. J. Phys. Chem. Lett. 2018, 9, 464–470. 10.1021/acs.jpclett.7b03316. 29320636

[ref7] Xia J.; Sanders S. N.; Cheng W.; Low J. Z.; Liu J.; Campos L. M.; Sun T. Singlet fission: progress and prospects in solar cells. Adv. Mater. 2017, 29, 1601652 10.1002/adma.201601652. 27973702

[ref8] Zimmerman P. M.; Musgrave C. B.; Head-Gordon M. A correlated electron view of singlet fission. Acc. Chem. Res. 2013, 46, 1339–1347. 10.1021/ar3001734. 23427823

[ref9] Bhattacharyya K.; Datta A. Polymorphism controlled singlet fission in TIPS-Anthracene: Role of stacking orientation. J. Phys. Chem. C 2017, 121, 1412–1420. 10.1021/acs.jpcc.6b10075.

[ref10] Müller U.; Roos L.; Frank M.; Deutsch M.; Hammer S.; Krumrein M.; Friedrich A.; Marder T. B.; Engels B.; Krueger A.; et al. Role of intermolecular interactions in the excited-state photophysics of tetracene and 2, 2’-ditetracene. J. Phys. Chem. C 2020, 124, 19435–19446. 10.1021/acs.jpcc.0c04066.

[ref11] Casanova D. Theoretical modeling of singlet fission. Chem. Rev. 2018, 118, 7164–7207. 10.1021/acs.chemrev.7b00601. 29648797

[ref12] Olsen J. M.; Aidas K.; Kongsted J. Excited States in Solution through Polarizable Embedding. J. Chem. Theory Comput. 2010, 6, 3721–3734. 10.1021/ct1003803.

[ref13] DeFusco A.; Minezawa N.; Slipchenko L. V.; Zahariev F.; Gordon M. S. Modeling Solvent Effects on Electronic Excited States. J. Phys. Chem. Lett. 2011, 2, 2184–2192. 10.1021/jz200947j.

[ref14] Lischka H.; Nachtigallová D.; Aquino A. J. A.; Szalay P. G.; Plasser F.; Machado F. B. C.; Barbatti M. Multireference Approaches for Excited States of Molecules. Chem. Rev. 2018, 118, 7293–7361. 10.1021/acs.chemrev.8b00244. 30040389

[ref15] Rocha-Rinza T.; Christiansen O. Linear response coupled cluster study of the benzene excimer. Chem. Phys. Lett. 2009, 482, 44–49. 10.1016/j.cplett.2009.09.088.

[ref16] Fradelos G.; Lutz J. J.; Wesołowski T. A.; Piecuch P.; Włoch M. Embedding vs Supermolecular Strategies in Evaluating the Hydrogen-Bonding-Induced Shifts of Excitation Energies. J. Chem. Theory Comput. 2011, 7, 1647–1666. 10.1021/ct200101x.

[ref17] Seifert G.; Joswig J.-O. Density-functional tight binding—an approximate density-functional theory method. Wiley Interdiscip. Rev.: Comput. Mol. Sci. 2012, 2, 456–465. 10.1002/wcms.1094.

[ref18] Briggs E. A.; Besley N. A. Modelling excited states of weakly bound complexes with density functional theory. Phys. Chem. Chem. Phys. 2014, 16, 14455–14462. 10.1039/C3CP55361B. 24531883

[ref19] Sato T.; Nakai H. Density functional method including weak interactions: Dispersion coefficients based on the local response approximation. J. Chem. Phys. 2009, 131, 224104 10.1063/1.3269802. 20001021

[ref20] Ikabata Y.; Nakai H. Extension of local response dispersion method to excited-state calculation based on time-dependent density functional theory. J. Chem. Phys. 2012, 137, 124106–124116. 10.1063/1.4754508. 23020323

[ref21] Pernal K. Electron Correlation from the Adiabatic Connection for Multireference Wave Functions. Phys. Rev. Lett. 2018, 120, 013001 10.1103/PhysRevLett.120.013001. 29350961

[ref22] Chatterjee K.; Pernal K. Excitation energies from extended random phase approximation employed with approximate one- and two-electron reduced density matrices. J. Chem. Phys. 2012, 137, 204109 10.1063/1.4766934. 23205983

[ref23] Hapka M.; Przybytek M.; Pernal K. Second-Order Exchange-Dispersion Energy Based on a Multireference Description of Monomers. J. Chem. Theory Comput. 2019, 15, 6712–6723. 10.1021/acs.jctc.9b00925. 31670950

[ref24] Hapka M.; Przybytek M.; Pernal K. Symmetry-Adapted Perturbation Theory Based on Multiconfigurational Wave Function Description of Monomers. J. Chem. Theory Comput. 2021, 17, 5538–5555. 10.1021/acs.jctc.1c00344. 34517707 PMC8444344

[ref25] Pastorczak E.; Pernal K. Correlation Energy from the Adiabatic Connection Formalism for Complete Active Space Wave Functions. J. Chem. Theory Comput. 2018, 14, 3493–3503. 10.1021/acs.jctc.8b00213. 29787257

[ref26] Pastorczak E.; Pernal K. Electronic Excited States from the Adiabatic-Connection Formalism with Complete Active Space Wave Functions. J. Phys. Chem. Lett. 2018, 9, 5534–5538. 10.1021/acs.jpclett.8b02391. 30192553

[ref27] Hapka M.; Pastorczak E.; Krzemińska A.; Pernal K. Long-range-corrected multiconfiguration density functional with the on-top pair density. J. Chem. Phys. 2020, 152, 094102 10.1063/1.5138980. 33480720

[ref28] Vydrov O. A.; Van Voorhis T. Nonlocal van der Waals density functional: The simpler the better. J. Chem. Phys. 2010, 133, 244103 10.1063/1.3521275. 21197972

[ref29] Klessinger M.; Michl J. Excited States and Photochemistry of Organic Molecules; VCH, 1995.

[ref30] Robb M. A.; Garavelli M.; Olivucci M.; Bernardi F. A Computational Strategy for Organic Photochemistry. Rev. Comput. Chem. 2000, 87–146. 10.1002/9780470125922.ch2.

[ref31] Reimers J. R.; Cai Z.-L. Hydrogen bonding and reactivity of water to azines in their S_1_ (*n, π**) electronic excited states in the gas phase and in solution. Phys. Chem. Chem. Phys. 2012, 14, 8791–8802. 10.1039/c2cp24040h. 22532059

[ref32] Ge Q.; Mao Y.; Head-Gordon M. Energy decomposition analysis for exciplexes using absolutely localized molecular orbitals. J. Chem. Phys. 2018, 148, 064105 10.1063/1.5017510. 29448791

[ref33] Xu Y.; Friedman R.; Wu W.; Su P. Understanding intermolecular interactions of large systems in ground state and excited state by using density functional based tight binding methods. J. Chem. Phys. 2021, 154, 194106 10.1063/5.0052060. 34240911

[ref34] Ikabata Y.; Nakai H. Extension of local response dispersion method to excited-state calculation based on time-dependent density functional theory. J. Chem. Phys. 2012, 137, 124106 10.1063/1.4754508. 23020323

[ref35] Grimme S.; Antony J.; Ehrlich S.; Krieg H. A consistent and accurate ab initio parametrization of density functional dispersion correction (DFT-D) for the 94 elements H-Pu. J. Chem. Phys. 2010, 132, 154104 10.1063/1.3382344. 20423165

[ref36] Řezáč J. Empirical Self-Consistent Correction for the Description of Hydrogen Bonds in DFTB3. J. Chem. Theory Comput. 2017, 13, 4804–4817. 10.1021/acs.jctc.7b00629. 28949517

[ref37] Horn P. R.; Mao Y.; Head-Gordon M. Defining the contributions of permanent electrostatics, Pauli repulsion, and dispersion in density functional theory calculations of intermolecular interaction energies. J. Chem. Phys. 2016, 144, 114107 10.1063/1.4942921. 27004862

[ref38] Jeziorski B.; Moszynski R.; Szalewicz K. Perturbation theory approach to intermolecular potential energy surfaces of van der Waals complexes. Chem. Rev. 1994, 94, 1887–1930. 10.1021/cr00031a008.

[ref39] Kaplan I. G. Intermolecular interactions: physical picture, computational methods and model potentials; John Wiley & Sons, 2006.

[ref40] Marinescu M.; Dalgarno A. Dispersion forces and long-range electronic transition dipole moments of alkali-metal dimer excited states. Phys. Rev. A 1995, 52, 311 10.1103/PhysRevA.52.311. 9912249

[ref41] Marinescu M. Dispersion coefficients for the nP-nP asymptote of homonuclear alkali-metal dimers. Phys. Rev. A 1997, 56, 4764 10.1103/PhysRevA.56.4764. 9910327

[ref42] Silvi B.; Chandrasekharan V. Dispersion coefficients for atoms in different states. Mol. Phys. 1983, 48, 1053–1066. 10.1080/00268978300100741.

[ref43] Casimir H. B. G.; Polder D. The Influence of Retardation on the London-van der Waals Forces. Phys. Rev. 1948, 73, 360–372. 10.1103/PhysRev.73.360.

[ref44] Longuet-Higgins H. Spiers memorial lecture. Intermolecular forces. Discuss. Faraday Soc. 1965, 40, 7–18. 10.1039/df9654000007.

[ref45] Dobson J. F.; Gould T. Calculation of dispersion energies. J. Phys.: Condens. Matter 2012, 24, 073201 10.1088/0953-8984/24/7/073201. 22213768

[ref46] Dobson J. In Time-Dependent Density Functional Theory; Marques M. A. L., et al., Eds.; Springer, 2006; pp 443–462.

[ref47] Power E.; Thirunamachandran T. Quantum electrodynamics with nonrelativistic sources. V. Electromagnetic field correlations and intermolecular interactions between molecules in either ground or excited states. Phys. Rev. A 1993, 47, 2539 10.1103/PhysRevA.47.2539. 9909221

[ref48] Hapka M.; Krzemińska A.; Pernal K. How Much Dispersion Energy Is Included in the Multiconfigurational Interaction Energy?. J. Chem. Theory Comput. 2020, 16, 6280–6293. 10.1021/acs.jctc.0c00681. 32877179 PMC7586340

[ref49] Pernal K. Exact and approximate adiabatic connection formulae for the correlation energy in multireference ground and excited states. J. Chem. Phys. 2018, 149, 204101 10.1063/1.5048988. 30501241

[ref50] Nguyen B. D.; Chen G. P.; Agee M. M.; Burow A. M.; Tang M. P.; Furche F. Divergence of many-body perturbation theory for noncovalent interactions of large molecules. J. Chem. Theory Comput. 2020, 16, 2258–2273. 10.1021/acs.jctc.9b01176. 32105488

[ref51] Parker T. M.; Burns L. A.; Parrish R. M.; Ryno A. G.; Sherrill C. D. Levels of symmetry adapted perturbation theory (SAPT). I. Efficiency and performance for interaction energies. J. Chem. Phys. 2014, 140, 094106 10.1063/1.4867135. 24606352

[ref52] Hapka M.; Przybytek M.; Pernal K. Second-Order Dispersion Energy Based on Multireference Description of Monomers. J. Chem. Theory Comput. 2019, 15, 1016–1027. 10.1021/acs.jctc.8b01058. 30525591

[ref53] Pernal K.; Chatterjee K.; Kowalski P. H. How accurate is the strongly orthogonal geminal theory in predicting excitation energies? Comparison of the extended random phase approximation and the linear response theory approaches. J. Chem. Phys. 2014, 140, 014101 10.1063/1.4855275. 24410215

[ref54] Pastorczak E.; Hapka M.; Veis L.; Pernal K. Capturing the Dynamic Correlation for Arbitrary Spin-Symmetry CASSCF Reference with Adiabatic Connection Approaches: Insights into the Electronic Structure of the Tetramethyleneethane Diradical. J. Phys. Chem. Lett. 2019, 10, 4668–4674. 10.1021/acs.jpclett.9b01582. 31356083

[ref55] Maradzike E.; Hapka M.; Pernal K.; DePrince A. E. Reduced Density Matrix-Driven Complete Active Apace Self-Consistent Field Corrected for Dynamic Correlation from the Adiabatic Connection. J. Chem. Theory Comput. 2020, 16, 4351–4360. 10.1021/acs.jctc.0c00324. 32538086

[ref56] Beran P.; Matoušek M.; Hapka M.; Pernal K.; Veis L. Density matrix renormalization group with dynamical correlation via adiabatic connection. J. Chem. Theory Comput. 2021, 17, 7575–7585. 10.1021/acs.jctc.1c00896. 34762423

[ref57] Pernal K. Intergeminal correction to the antisymmetrized product of strongly orthogonal geminals derived from the extended random phase approximation. J. Chem. Theory Comput. 2014, 10, 4332–4341. 10.1021/ct500478t. 26588130

[ref58] Toulouse J.; Zhu W.; Angyán J. G.; Savin A. Range-separated density-functional theory with the random-phase approximation: Detailed formalism and illustrative applications. Phys. Rev. A 2010, 82, 032502 10.1103/PhysRevA.82.032502. 20590182

[ref59] Goll E.; Werner H.-J.; Stoll H. A short-range gradient-corrected density functional in long-range coupled-cluster calculations for rare gas dimers. Phys. Chem. Chem. Phys. 2005, 7, 3917–3923. 10.1039/b509242f. 19810319

[ref60] Murrell J. N.; Randić M.; Williams D. The theory of intermolecular forces in the region of small orbital overlap. Proc. R. Soc. A 1965, 284, 566–581. 10.1098/rspa.1965.0081.

[ref61] Jeziorska M.; Jeziorski B.; Čížek J. Direct calculation of the Hartree–Fock interaction energy via exchange–perturbation expansion. The He ··· He interaction. Int. J. Quantum Chem. 1987, 32, 149–164. 10.1002/qua.560320202.

[ref62] Moszyński R.; Heijmen T.; Jeziorski B. Symmetry-adapted perturbation theory for the calculation of Hartree-Fock interaction energies. Mol. Phys. 1996, 88, 741–758. 10.1080/00268979609482451.

[ref63] Misquitta A. J.; Podeszwa R.; Jeziorski B.; Szalewicz K. Intermolecular potentials based on symmetry-adapted perturbation theory with dispersion energies from time-dependent density-functional calculations. J. Chem. Phys. 2005, 123, 214103 10.1063/1.2135288. 16356035

[ref64] Hesselmann A.; Jansen G.; Schütz M. Density-functional theory-symmetry-adapted intermolecular perturbation theory with density fitting: A new efficient method to study intermolecular interaction energies. J. Chem. Phys. 2005, 122, 014103 10.1063/1.1824898. 15638638

[ref65] Langreth D. C.; Lundqvist B. I.; Chakarova-Käck S. D.; Cooper V. R.; Dion M.; Hyldgaard P.; Kelkkanen A.; Kleis J.; Kong L.; Li S.; Moses P. G.; Murray E.; Puzder A.; Rydberg H.; Schröder E.; Thonhauser T. A density functional for sparse matter. J. Phys.: Condens. Matter 2009, 21, 084203 10.1088/0953-8984/21/8/084203. 21817355

[ref66] Vydrov O. A.; Voorhis T. V. In Fundamentals of Time-Dependent Density Functional Theory; Marques M. A., Maitra N. T., Nogueira F. M., Gross E., Rubio A., Eds.; Springer Berlin Heidelberg: Berlin, Heidelberg, 2012; pp 443–456.

[ref67] Hujo W.; Grimme S. Performance of the van der Waals Density Functional VV10 and (hybrid)GGA Variants for Thermochemistry and Noncovalent Interactions. J. Chem. Theory Comput. 2011, 7, 3866–3871. 10.1021/ct200644w. 26598333

[ref68] Grimme S.; Hansen A.; Brandenburg J. G.; Bannwarth C. Dispersion-Corrected Mean-Field Electronic Structure Methods. Chem. Rev. 2016, 116, 5105–5154. 10.1021/acs.chemrev.5b00533. 27077966

[ref69] Shahbaz M.; Szalewicz K. Dispersion Energy from Local Polarizability Density. Phys. Rev. Lett. 2019, 122, 213001 10.1103/PhysRevLett.122.213001. 31283348

[ref70] Murray E. D.; Lee K.; Langreth D. C. Investigation of Exchange Energy Density Functional Accuracy for Interacting Molecules. J. Chem. Theory Comput. 2009, 5, 2754–2762. 10.1021/ct900365q. 26631788

[ref71] Perdew J. P.; Burke K.; Ernzerhof M. Generalized Gradient Approximation Made Simple. Phys. Rev. Lett. 1996, 77, 3865–3868. 10.1103/PhysRevLett.77.3865. 10062328

[ref72] Pernal K.; Podeszwa R.; Patkowski K.; Szalewicz K. Dispersionless Density Functional Theory. Phys. Rev. Lett. 2009, 103, 263201 10.1103/PhysRevLett.103.263201. 20366310

[ref73] Rajchel L.; Żuchowski P. S.; Szczȩśniak M. M.; Chałasiński G. Density Functional Theory Approach to Noncovalent Interactions via Monomer Polarization and Pauli Blockade. Phys. Rev. Lett. 2010, 104, 163001 10.1103/PhysRevLett.104.163001. 20482044

[ref74] Řezáč J.; Riley K. E.; Hobza P. S66: A Well-balanced Database of Benchmark Interaction Energies Relevant to Biomolecular Structures. J. Chem. Theory Comput. 2011, 7, 2427–2438. 10.1021/ct2002946. 21836824 PMC3152974

[ref75] Řezáč J.; Riley K. E.; Hobza P. Erratum to “S66: A Well-balanced Database of Benchmark Interaction Energies Relevant to Biomolecular Structures. J. Chem. Theory Comput. 2014, 10, 1359–1360. 10.1021/ct5000692. 26580199 PMC3954041

[ref76] Boys S.; Bernardi F. The calculation of small molecular interactions by the differences of separate total energies. Some procedures with reduced errors. Mol. Phys. 1970, 19, 553–566. 10.1080/00268977000101561.

[ref77] Monkhorst H. J. Calculation of properties with the coupled-cluster method. Int. J. Quantum Chem. 1977, 12, 421–432. 10.1002/qua.560120850.

[ref78] Krishnan R.; Binkley J. S.; Seeger R.; Pople J. A. Self-consistent molecular orbital methods. XX. A basis set for correlated wave functions. J. Chem. Phys. 1980, 72, 650 10.1063/1.438955.

[ref79] Clark T.; Chandrasekhar J.; Spitznagel G. W.; Schleyer P. V. R. Efficient diffuse function-augmented basis-sets for anion calculations. 3. The 3-21+G basis set for 1st-row elements, Li-F. J. Comput. Chem. 1983, 4, 294–301. 10.1002/jcc.540040303.

[ref80] Frisch M. J.; Pople J. A.; Binkley J. S. Self-Consistent Molecular Orbital Methods. 25. Supplementary Functions for Gaussian Basis Sets. J. Chem. Phys. 1984, 80, 3265–3269. 10.1063/1.447079.

[ref81] Kendall R.; Dunning A. T. H. Jr.; Harrison R. J. Electron affinities of the first-row atoms revisited. Systematic basis sets and wave functions. J. Chem. Phys. 1992, 96, 6796–6806. 10.1063/1.462569.

[ref82] Werner H.-J.; Knowles P. J.; Knizia G.; Manby F. R.; Schütz M. Molpro: a general-purpose quantum chemistry program package. WIREs Comp. Mol. Sci. 2012, 2, 242 10.1002/wcms.82.

[ref83] Sharma P.; Bernales V.; Truhlar D. G.; Gagliardi L. Valence *ππ** Excitations in Benzene Studied by Multiconfiguration Pair-Density Functional Theory. J. Phys. Chem. Lett. 2019, 10, 75–81. 10.1021/acs.jpclett.8b03277. 30540476

[ref84] Lorentzon J.; Fülscher M. P.; Roos B. A theoretical study of the electronic spectra of pyridine and phosphabenzene. Theor. Chim. Acta 1995, 92, 67–81. 10.1007/BF01134214.

[ref85] Besley N. A.; Hirst J. D. Ab Initio Study of the Effect of Solvation on the Electronic Spectra of Formamide and N-Methylacetamide. J. Phys. Chem. A 1998, 102, 10791–10797. 10.1021/jp982645f.

[ref86] Pernal K.; Hapka M. GammCor code. https://github.com/pernalk/GAMMCOR (accessed 2021).

[ref87] Celani P.; Werner H.-J. Multireference perturbation theory for large restricted and selected active space reference wave functions. J. Chem. Phys. 2000, 112, 5546–5557. 10.1063/1.481132.

[ref88] Ikabata Y.; Nakai H. Local response dispersion method: A density-dependent dispersion correction for density functional theory. Int. J. Quantum Chem. 2015, 115, 309–324. 10.1002/qua.24786.

[ref89] Taylor D. E.; Ángyán J. G.; Galli G.; Zhang C.; Gygi F.; Hirao K.; Song J. W.; Rahul K.; Anatole von Lilienfeld O.; Podeszwa R.; Bulik I. W.; Henderson T. M.; Scuseria G. E.; Toulouse J.; Peverati R.; Truhlar D. G.; Szalewicz K. Blind test of density-functional-based methods on intermolecular interaction energies. J. Chem. Phys. 2016, 145, 124105 10.1063/1.4961095. 27782652

