# Supplementary material for: Dispersion Interactions in Exciton-Localized States. Theory and Applications to π–π* and n−π* Excited States
Source: J Chem Theory Comput. 2022 May 19;18(6):3497–511. doi: 10.1021/acs.jctc.2c00221 (PMC9202351; doi:10.1021/acs.jctc.2c00221)
Supplement: Supplementary file 1 — ct2c00221_si_001.pdf [file ct2c00221_si_001.pdf]

Supporting Information for

Dispersion interactions in exciton-localised states. Theory and applications to $\pi - \pi^*$ and $n - \pi^*$ excited states.

Mohammad Reza Jangrouei,[1] Agnieszka Krzemińska,[1] Michał Hapka,[2] Ewa Pastorczak,[1] and Katarzyna Pernal[1, a)]

[1)] *Institute of Physics, Lodz University of Technology, ul. Wolczanska 217/221, 93-005 Lodz, Poland*

[2)] *Faculty of Chemistry, University of Warsaw, ul. L. Pasteura 1, 02-093 Warsaw, Poland*

[a)] Electronic mail: pernalk@gmail.com

I. REPARAMETERIZATION OF THE VV10 CORRELATION FUNCTIONAL

VV10 functional has been reparameterized by minimizing the weighted mean absolute percentage error defined as

$$M = \frac{100}{\sum_{n=1}^{s} p_n} \sum_{n=1}^{s} W_n \sum_{t=1}^{p_n} \left| \frac{A_t - F_t}{A_t} \right| \quad . \tag{1}$$

A_t is SAPT(DFT) dispersion energy from Ref. 1, F_t is VV10 interaction correlation energy calculated according to Eq.(58) [see main text], n pertains to a dimer, W_n is the optimization weight, and p_n is the number of data point of the dimer and includes the short, medium, and long range of the molecular distances. Argon dimer is described with $n = 1$, $W_1 = 1$, and $p_1 = 11$, Ethanol dimer with $n = 2$, $W_2 = 3$, and $p_2 = 7$, and Water dimer with $n = 3$, $W_3 = 1$ and $p_3 = 16$.

The molecular structures are taken from Ref. 2 and the input electron densities are calculated using Molpro package[3] with PBE exchange-correlation functional and aug-cc-pVTZ basis set. Nelder–Mead method (downhill simplex algorithm)[4] is employed to optimize VV10 parameters. For the training set the mean absolute percentage error (MAPE) of 11% and the mean error (ME) of 0.37 mHa have been achieved.

TABLE S1. MAPEs and MEs of the dispersion energies estimated by the reparameterized VV10[5] (reVV10) and damped asymptotic dispersion energy[1] (DADE) nonlocal correlation functionals with respect to SAPT(DFT)[67] benchmarks. The PBE exchange-correlation functional[8] is used to calculate the input densities with the aug-cc-pVTZ basis sets.

	Data points	MAPE[%]		ME[mHa]	
		reVV10	DADE	reVV10	DADE
Ar-Ar	11	8.69	9.55	0.02	0.04
Water-Water	16	12.94	15.03	0.82	0.45
Ethanol-Ethanol	7	10.08	6.77	−0.13	−0.11
Ar-HF	9	6.44	6.86	0.02	0.03
Nitromethane dimer	7	8.35	10.52	−0.19	0.43
Methylformate dimer	6	7.19	6.85	0.09	0.08
Benzene-Methane	7	7.01	6.58	−0.05	−0.08
Benzene-Water	9	30.68	9.06	0.09	−0.02
Imidazole dimer	7	12.40	24.89	1.23	0.62
Nitrobenzene dimer	7	21.67	7.82	−1.90	−0.57
Ethylenedinitramine dimer	8	14.61	11.55	−0.90	0.46
Total	94	12.91	9.80	0.004	0.15

TABLE S2. Interaction energy components of SAPT(CAS) and their sums ($E_{\mathrm{int}}^{\mathrm{SAPT}}$) obtained for ground state complexes shown in Figure 1 in the main text [where the visualization software from Ref. 9 has been utilized]. Own calculations, see the main text. All values are reported in kcal·mol^{-1}.

	$E_{\mathrm{elst}}^{(1)}$	$E_{\mathrm{exch}}^{(1)}$	$E_{\mathrm{ind}}^{(2)}$	$E_{\mathrm{exch-ind}}^{(2)}$	$E_{\mathrm{disp}}^{(2)}$	$E_{\mathrm{exch-disp}}^{(2)}$	$E_{\mathrm{int}}^{\mathrm{SAPT}}$
benzene-water	−2.74	3.16	−1.33	0.70	−3.06	0.37	−2.89
benzene-MeOH	−3.08	4.52	−1.72	1.04	−4.89	0.58	−3.55
benzene-$MeNH_2$	−2.22	3.98	−1.20	0.91	−4.86	0.59	−2.79
pyridine-water	−11.19	10.64	−5.18	2.95	−4.09	0.84	−6.04
pyridine-MeOH	−11.76	11.77	−5.94	3.52	−5.00	0.99	−6.42
pyridine-$MeNH_2$	−4.06	5.61	−1.83	1.33	−5.17	0.69	−3.42
peptide-water	−6.71	5.36	−2.07	1.04	−2.93	0.45	−4.87
peptide-$MeNH_2$	−10.55	10.86	−4.92	3.03	−5.70	1.05	−6.24

TABLE S3. AC0 correlation energy contributions to interaction energies for the ground state (upper part) and $\pi-\pi^*$ (benzene and pyridine complexes) and $n-\pi^*$ (peptide complexes) excited state (lower part) systems confronted with the coupled and uncoupled (UC) dispersion energy $E_{\rm DISP}$ given as a sum of $E^{(2)}_{\rm disp}$ and exchange-dispersion $E^{(2)}_{\rm exch-disp}$ terms, see Eq.(46). Own calculations, see the main text. Values are reported in kcal·mol^{-1}.

ground state	AC0	$E^{\rm UC}_{\rm DISP}$	$E_{\rm DISP}$
benzene-water	−2.46	−2.85	−2.68
benzene-MeOH	−4.04	−4.60	−4.29
benzene-$MeNH_2$	−4.05	−4.60	−4.25
pyridine-water	−2.01	−3.10	−3.24
pyridine-MeOH	−2.73	−3.89	−4.00
pyridine-$MeNH_2$	−4.15	−4.73	−4.46
peptide-water	−1.76	−2.43	−2.47
peptide-$MeNH_2$	−3.84	−4.67	−4.64
excited state	AC0	$E^{\rm UC}_{\rm DISP}$	$E_{\rm DISP}$
benzene-water	−2.50	−2.73	−2.55
benzene-MeOH	−3.58	−4.42	−4.11
benzene-$MeNH_2$	−3.91	−4.43	−4.08
pyridine-water	−2.41	−3.08	−3.21
pyridine-MeOH	−2.71	−3.85	−3.96
pyridine-$MeNH_2$	−4.04	−4.60	−4.34
peptide-water	−2.00	−2.46	−2.47
peptide-$MeNH_2$	−4.10	−4.77	−4.69

REFERENCES

[1]M. Shahbaz and K. Szalewicz, "Dispersion energy from local polarizability density," Phys. Rev. Lett. **122**, 213001 (2019).

[2]D. E. Taylor, J. G. Ángyán, G. Galli, C. Zhang, F. Gygi, K. Hirao, J. W. Song, K. Rahul, O. Anatole von Lilienfeld, R. Podeszwa, I. W. Bulik, T. M. Henderson, G. E. Scuseria, J. Toulouse, R. Peverati, D. G. Truhlar, and K. Szalewicz, "Blind test of density-functional-based methods on intermolecular interaction energies," J. Chem. Phys. **145**, 124105 (2016).

[3]H.-J. Werner, P. J. Knowles, G. Knizia, F. R. Manby, and M. Schütz, "Molpro: a general-purpose quantum chemistry program package," (2012).

[4]J. A. Nelder and R. Mead, "A Simplex Method for Function Minimization," The Computer Journal **7**, 308–313 (1965).

[5]O. A. Vydrov and T. Van Voorhis, "Nonlocal van der waals density functional: The simpler the better," J. Chem. Phys. **133**, 244103 (2010).

[6]A. J. Misquitta, R. Podeszwa, B. Jeziorski, and K. Szalewicz, "Intermolecular potentials based on symmetry-adapted perturbation theory with dispersion energies from time-dependent density-functional calculations," J. Chem. Phys. **123**, 214103 (2005).

[7]A. Hesselmann, G. Jansen, and M. Schütz, "Density-functional theory-symmetry-adapted intermolecular perturbation theory with density fitting: A new efficient method to study intermolecular interaction energies," J. Chem. Phys. **122**, 014103 (2005).

[8]J. P. Perdew, K. Burke, and M. Ernzerhof, "Generalized gradient approximation made simple," Phys. Rev. Lett. **77**, 3865–3868 (1996).

[9]P. H. Kowalski, "General visualization tool," `https://github.com/hemiku/Visualization`, last accessed on 26/01/22.
